# Comparative transcriptome analysis reveals molecular damage associated with cryopreservation in *Crassostrea angulata* D-larvae rather than to cryoprotectant exposure

**DOI:** 10.1186/s12864-024-10473-1

**Published:** 2024-06-12

**Authors:** Catarina Anjos, Daniel Duarte, Elvira Fatsini, Domitília Matias, Elsa Cabrita

**Affiliations:** 1 Centre of Marine Sciences-CCMAR/CIMAR.LA, University of Algarve, Faro, 8005-139 Portugal; 2 Portuguese Institute for Sea and Atmosphere-IPMA, Av. 5 de Outubro, Olhão, 8700-305 Portugal

**Keywords:** Portuguese oyster, Cryoprotectant exposure, D-larvae cryopreservation, Cryodamage, RNA-seq, Gene expression

## Abstract

**Background:**

The Portuguese oyster *Crassostrea angulata*, a bivalve of significant economic and ecological importance, has faced a decline in both production and natural populations due to pathologies, climate change, and anthropogenic factors. To safeguard its genetic diversity and improve reproductive management, cryopreservation emerges as a valuable strategy. However, the cryopreservation methodologies lead to some damage in structures and functions of the cells and tissues that can affect post-thaw quality. Transcriptomics may help to understand the molecular consequences related to cryopreservation steps and therefore to identify different freezability biomarkers. This study investigates the molecular damage induced by cryopreservation in *C. angulata* D-larvae, focusing on two critical steps: exposure to cryoprotectant solution and the freezing/thawing process.

**Results:**

Expression analysis revealed 3 differentially expressed genes between larvae exposed to cryoprotectant solution and fresh larvae and 611 differentially expressed genes in cryopreserved larvae against fresh larvae. The most significantly enriched gene ontology terms were “carbohydrate metabolic process”, “integral component of membrane” and “chitin binding” for biological processes, cellular components and molecular functions, respectively. Kyoto Encyclopedia of Genes and Genomes enrichment analysis identified the “neuroactive ligand receptor interaction”, “endocytosis” and “spliceosome” as the most enriched pathways. RNA sequencing results were validate by quantitative RT-PCR, once both techniques presented the same gene expression tendency and a group of 11 genes were considered important molecular biomarkers to be used in further studies for the evaluation of cryodamage.

**Conclusions:**

The current work provided valuable insights into the molecular repercussions of cryopreservation on D-larvae of *Crassostrea angulata*, revealing that the freezing process had a more pronounced impact on larval quality compared to any potential cryoprotectant-induced toxicity. Additionally, was identify 11 genes serving as biomarkers of freezability for D-larvae quality assessment. This research contributes to the development of more effective cryopreservation protocols and detection methods for cryodamage in this species.

## Background

The Portuguese oyster *Crassostrea angulata*, a bivalve of major economic value and widely distributed around the world [1], used to be an important resource for the European aquaculture industry until the 1970s. By this decade it suffered a great mortality mainly due to an iridovirus disease [2] and since then, not only its production but also its natural populations are endangered due to climate and anthropogenic factors [3]. To overcome this situation, there is a need to create tools and strategies to preserve this species natural banks and to improve its production [4].

Cryopreservation presents itself as a valuable strategy to secure the storage of important genetic lines of endangered species, preserving biodiversity, and to improve the management of a species reproduction [5]. Many studies show the possibility of cryopreserving both gametes and larvae of different invertebrates, including bivalve species such as oysters [6–8]. When compared to sperm storage, the main advantage of larvae cryopreservation is the immediate availability of a diploid organism upon thawing [9, 10]. However, there are some challenges on the cryopreservation of larvae when compared to gametes, such as the size and complexity of a multicellular organism [10, 11].

The improvement of cryopreservation methodologies is imperative to obtain high post-thaw larvae quality. For that purpose, the evaluation of the effect of cryopreservation steps, such as cryoprotectants solutions and freezing process, on the biological processes and pathways of the organism is essential for the establishment of a reliable protocol [12].

The most common quality parameters used to evaluate larval post-thaw quality are swimming activity, morphology, and survival [8]. Information is still scarce, but new technologies are finding their way to become valuable tools in this type of studies. From a molecular point of view, the use of techniques such as transcriptomic and proteomic analysis in cryobiology [13–15], may help to identify different freezability biomarkers and to understand the molecular consequences related to cryopreservation. This will be extremely important for a species such *C. angulata*, where cryopreservation tools can be applied for the creation of genebanks to secure species preservation.

The application of transcriptome analysis technologies such as next-generation sequencing (NGS) is becoming widespread, using tools such as RNA sequencing (RNA-seq) that allow gene identification and their respective expression [16, 17]. Gene expression have been successfully used in the investigation of sperm and larvae cryodamage in several species including bivalves [18–21]. There are studies in the use of RNA-seq to identify gene alterations in post-thaw sperm and embryos of different species [22–24]. In blue catfish (*Ictalurus punctatus*) cryopreserved sperm, authors identified an upregulation in genes related to sperm motility-related functions (cilium, motile cilium, and microtubule cytoskeleton) and amide (often used as a cryoprotectant in sperm preservation) biosynthesis pathway [23]. In kelp grouper cryopreserved larvae (*Epinephelus moara*) it was identified a downregulation of genes related to eye development, cranial nerve development, sensory light stimulation and neurotransmitter transport, suggesting an impairment of larvae central nervous system development [22]. However, information is still scarce regarding invertebrate larvae and the effects are only associated to the last step of cryopreservation not taking into account the possible toxicity of cryoprotectants during exposure. In a previous study performed by our group in cryopreserved *C. angulata* D-larvae it was identified that the use of different cryoprotectant solutions induced an increase in larvae abnormalities incidence and a reduction of larvae swimming velocity after thawing [25]. However, there is no information about more in-depth damage resulted from gene alterations that can compromise further survival and development. The use of transcriptomic tools would be useful to investigate the molecular damage induced by all steps of the cryopreservation process and to identify putative cryodamage biomarkers on oyster´s larvae. This can be achieved by the full screening of the alterations of larvae molecular networks and biological processes promoted by cryopreservation. Therefore, this comprehensive investigation would support the selection of the most successful cryopreservation methodologies and potentially the identification of new analytical methods for the detection of relevant cryodamage in this species.

The objective of this study was to characterize the molecular damage promoted by cryopreservation in *C. angulata* D-larvae, using transcriptomic tools in two critical cryopreservation steps namely exposure to cryoprotectant solution (CPAs) and freezing/thawing process. Additionally, this work aims to identify putative quality biomarkers to understand the potential impact in larval structures, biological, cellular and molecular functions.

## Methods

### Biological material

*C. angulata* breeders were acquired from Neptunpearl Lda. bivalve farm (Setúbal, Portugal) during their natural spawning period, between May and July. These individuals were transported to IPMA Experimental Station of Shellfish Production (Tavira, Portugal) and kept at 4 ºC for a maximum of 24 h. Prior to gametes collection, the oysters were wiped to removed debris and fouling organisms.

### Gamete collection and D-larvae production

The oysters were opened, and their sex was determined by microscopic observation of gametes obtained by a small incision in the gonad. Each breeder was independently stripped to collect the gametes for posterior fertilization. Egg suspensions were filtered at 100 μm and retain in 20 μm mesh screen, and spermatozoa sieved at 20 μm, to reduce potential contaminations. Both gametes were maintained in filtered and UV- sterilized seawater (FSW), until the fertilization. The sperm motility and concentration were evaluated by computer-assisted sperm analysis (CASA) system (Proiser R + D S.L., Valencia, Spain), while the oocyte sphericity and concentration were confirmed after 20 min of contact with FSW through observation via light microscopy. Only males with motile spermatozoa and females with spherical oocytes were used. A total of three males and three females were crossed to produce each larvae pool. The fertilization was carried out with a spermatozoon to oocyte proportion of 1000:1. One hour after fertilization, the eggs were filtered at a 20 μm mesh screen, to remove the remaining sperm, and incubated at 21 ± 1 °C for 24 to 30 h until reaching the D-larvae stage. Each pool was incubated in a 250 L tank with FSW at a concentration of 100 eggs per mL, with slight aeration. The larvae were recovered at a 30 μm mesh screen and kept in FSW to determine their concentration. The total number of larvae in each pool was counted in triplicates under microscopic observation, using a Sedgewick Rafter counting chamber. Pooled D-larvae with no apparent malformations were concentrated (around 120,000 larvae per mL) in FSW and kept on ice (4 °C) for a maximum of 2 h. A total of three pools (*n* = 3) were obtained in this study.

### Experimental design

To characterize the transcriptional changes related with the different steps of cryopreservation, the whole transcriptome profile of fresh larvae diluted in FSW (fresh larvae), fresh larvae exposed to a CPAs (cryoprotectant exposed larvae) and post-thaw larvae (cryopreserved larvae) was compared.

For this purpose, each D-larvae pool was exposed to the three following conditions, one control group and two treatments. As a control group, fresh larvae were concentrated in FSW as previously described. In the first treatment, larvae exposed to CPAs, the concentrated D-larvae were diluted with a 1:1 proportion in a CPAs consisting in a final concentration of 10% (v/v) dimethyl sulfoxide (DMSO) (Sigma-Aldrich), 1% (w/v) Polyvinylpyrrolidone (PVP-40) 40,000 MW (Sigma-Aldrich) and 0.2 M Sucrose (Sigma-Aldrich) in milli-Q water. Larvae were incubated in the cryoprotectant solution for 3 min (equilibrium time) at 4 ºC, since in a preliminary trial higher exposure time interfered with larval movement pattern. After incubation, the pools of D-larvae were diluted in a 1:3 proportion in FSW to dissipate the CPAs. The second treatment intended to evaluate the effects of the freezing/thawing process using a cryoprotectant solution based on DMSO. DMSO was chosen to see any further implications at molecular level in *Crassostrea angulata* larval cryopreservation. Following the same procedure as in the first treatment, the pooled larvae were incubated with the same CPAs. During the equilibrium time, the larvae pools diluted in the CPAs were loaded to 0.5 mL French straws (30,000 per straw) and maintained at 4 °C until finishes the 3 min equilibrium time. Subsequently, the straws were frozen in a programable biofreezer (Asymptote Grant EF600, United Kingdom) according to [9] with the following freezing protocol: 2.5 °C/min from 0 to -10 °C, hold for 5 min at -10 °C, 0.3 °C/min from − 10 °C to -20 °C and 2.5 °C/min down to -35 °C, and finally, plunged into liquid nitrogen (LN) and stored in a LN container. After two months of storage, straws were thawed in a water bath set at 37 °C for 10 s. Afterward, a recovery bath was prepared, by diluting the content of each straw in 2 L of FSW during a period of incubation of 1 h at room temperature. This procedure allowed the cryoprotectant dissipation. The post-thaw D-larvae were collected in a 30 μm mesh screen, washed and concentrated in 1.5 mL of FSW for further analyses.

### Transcriptome analysis

#### RNA extraction, library preparation and sequencing

Whole transcriptome analysis was performed in triplicate (*n* = 3) for pools of the fresh larvae, cryoprotectant exposed larvae and cryopreserved larvae. For this purpose, pools of 30,000 D-larvae were centrifuged at 7,400 g for 5 min at 4 °C to remove the FSW. Larvae pellets were then resuspended in 1 mL of TRI Reagent® (Sigma-Aldrich) and stored at -80 °C, until the RNA extraction.

RNA was isolated using TRI Reagent® (Sigma-Aldrich), according to the manufacturer’s recommendations, and total RNA was posteriorly purified using the NucleoSpin® RNA II kit (Macherey-Nagel, Germany). One treatment of dsDNase was performed to avoid genomic DNA contamination. The concentration and purity of the total RNA samples were measured using a NanoDrop One spectrophotometer (Thermo Fisher Scientific, USA). The RNA integrity was assessed on a bioanalyzer in RIN value (RNA integrity number ≥ 8.6), assessed by Experion RNA StdSens analysis kit (BIO-RAD). A total of 9 RNA samples were stored at -80 °C, for further analyses.

Whole transcriptome sequencing and respective bioinformatic analysis were performed by the Lifesequencing S.L.-ADM company (Valencia, Spain).

A total of 9 libraries were prepared starting from 1 µg of total RNA using a TruSeq RNA Library Preparation Kit v2 of Illumina (Illumina, USA). To confirm the quality of these libraries (library size: 276–348 bp; concentration: 76–162 nM) a HS D5000 Kit of Agilent 4200 was used, in a TapeStation bioanalyzer (Agilent technologies, USA). The libraries were sequenced in the NovaSeq 6000 Illumina instrument generating paired-end 150 bases reads.

#### Reads processing, mapping, and annotation

The raw reads were filtered using the BBTols v38.75 software (Bushnell B.). The sequencing adapters, low quality sequences (< Q20) and short sequences (< 40 nucleotides) were removed. Additionally, the reads were checked for potential contamination of bacteria, eukaryote, or archaea rRNA with the SortMeRNA v2.1b program [26]. As *C. angulata* whole genome was not available [27], the clean reads were then aligned to the reference genome of *Crassostrea gigas* (RefSeq accession: GCF_000297895.1 from the NCBI database). The proteins were then functionally annotated with the gene ontology (GO) terms by Blast against the Bivalvia taxa (taxID 6544) proteins, using OmicsBox v1.2 program [28]. Due to the presence of many isoforms, the quantification of the expression was taken to the gene level, a step performed by the Salmon v1.1 software [29].

### Differential expression analysis

Differences in expression were assessed using the DESeq2 v3.10 R package [30]. Counts were then filtered to remove the unexpressed genes and those with an expression lower than 5 counts in at least 3 samples. Thresholds were set for significant differential expression as False Discovery Rate (FDR) < 0.05 and │log_2_FC│> 1.5 (FC – Fold Change) for all the comparisons.

#### Gene set enrichment analysis (GSEA)

The functional enrichment analysis was carried out with all the genes and not only those with significant different expression. GO terms with 10–600 genes annotated and 1,000 permutations were the conditions chosen for the GSEA. The threshold defined for the results was a FDR of 0.05.

#### RT-qPCR confirmation

To confirm our RNA-seq data and define putative cryodamage markers in *C. angulata* cryopreservation, eleven differentially expressed genes (DEGs) were selected for quantitative RT-PCR (RT-qPCR). The genes were selected for their relation to the oyster growth (*adgre3*, dynein beta), structure-mantle and shell formation- (*mp, fbn2, myob3b*), oxidative stress (*epx, hsp70*) and immune response (*bp10, muc19, socs5*, Lectin). The 18 S gene was used as reference gene. The primers were designed using Primer-Blast (NCBI) (Table 1). Eight hundred nanograms of total RNA were reverse-transcribed to cDNA using a Maxima™ First Strand cDNA Synthesis Kit for RT-qPCR with dsDNase (Thermo Scientific™), following the manufacturer instructions. The qPCR reactions were conducted in duplicate on a CFX96 realtime PCR Detection System (Bio-Rad Laboratories, Hercules, CA) using a SsoFast™ EvaGreen® Supermix. In a total volume of 15 µL, the PCR reaction contained 7.5 µL of supermix, 0.75 µL of each 10 µM forward and reverse primers, and 3.75 µL of cDNA. The thermo cycling protocol used was as follow: 3 min at 95 ºC for an initial denaturation followed by 30 cycles of denaturation at 95 ºC for 30 s, annealing at 57 ºC for 30 s and extending at 72 ºC for 1 min. A final extension step was carried at 72 ºC for 5 min. To normalize the data, the expression of the reference gene in a pool of all samples was used. The relative expression of transcripts was evaluated by the 2^ΔΔCt^ method [31].

**Table 1:** List of forward (FW) and reverse (RV) primers used for the different transcripts analyzed through quantitative RT-PCR

Gene	Primer	
adhesion G protein-coupled receptor E3-like	**FW**	GCCTGAGTATGGCGTTGGAT
	**RV**	TAAGCACCCGGGACGTTTTT
blastula protease 10	**FW**	TATATCCCTCCGCCCAGGAC
	**RV**	CTGAGGTTTCGCAACGGTCT
eosinophil peroxidase	**FW**	CCCAGGAGACTGTACGGAGA
	**RV**	TCGGGAGGCAGTCAACTCTA
heat shock protein 70 B2	**FW**	GCGCACTCAAACGACGAAAA
	**RV**	CCGTGTCTGTGAATGCAACG
mucin-19	**FW**	GAGGTGCCGGAATAGCTCCA
	**RV**	ATGCGCTCATTGCGTTGTCA
mantle protein	**FW**	ACCCCGTCGATGTTACCAAG
	**RV**	CCTTTGGATTCGTAACCGCC
suppressor of cytokine signaling 5-like	**FW**	AGTCAGCTTCCGGCGATATG
	**RV**	TGTACGATGCAAGGGAGTGG
fibrillin-2	**FW**	CGGAGGATTTCGATGTGAGT
	**RV**	TGAATACCCTTCCCAACAGC
dynein beta chain X5	**FW**	AAAGTGACCACTCTCAGCAGC
	**RV**	GCATTATCTGTCCAGTGTCCTCA
myosin-IIIb	**FW**	TCCGACCAGAAAAATTCTAGCCA
	**RV**	GGAATAGGCTTGGCCACTGA
lectin	**FW**	GCTCTCCTGGTGGGACTTTT
	**RV**	TCGTTGGCTGCATCTGAACA
18 S	**FW**	GTCTGGTTAATTCCGATAACGAACGGAACTCTA
	**RV**	TGCTCAATCTCGTGTGGCTAAACGCAACTTG

## Results

### RNA sequencing and mapping

A pair end sequencing of 9 libraries was performed using a NovaSeq 6000 Illumina instrument. After a filtering step, 375,187,599 total clean reads were obtained. The average quality of the generated reads ranged from 35.50 to 36.18. The distribution of raw and clean reads among the different samples, as well as their mapping rate, are represented in the Table 2.

**Table 2:** RNA sequencing results for the reads counting and mapping to the reference genome (*Crassostrea gigas*) in each replicate of the three different treatments: Fresh (fresh larvae diluted in FSW), CPA exposure (fresh larvae exposed to CPAs) and Cryopreserved (post-thaw larvae)

Sample name	Raw reads	Clean reads	Mapping rate
Fresh 1	39,715,992	35,869,429	78.26%
Fresh 2	37,386,071	34,327,775	71.32%
Fresh 3	62,447,666	57,490,869	78.36%
CPA exposure 1	54,670,784	48,589,140	78.67%
CPA exposure 2	48,127,214	44,519,020	75.57%
CPA exposure 3	62,107,727	57,668,375	79.75%
Cryopreserved 1	33,792,962	30,215,425	74.03%
Cryopreserved 2	38,604,604	34,370,367	66.38%
Cryopreserved 3	36,055,724	32,137,199	76.05%

### Differential expression analysis

**Fig. 1:**
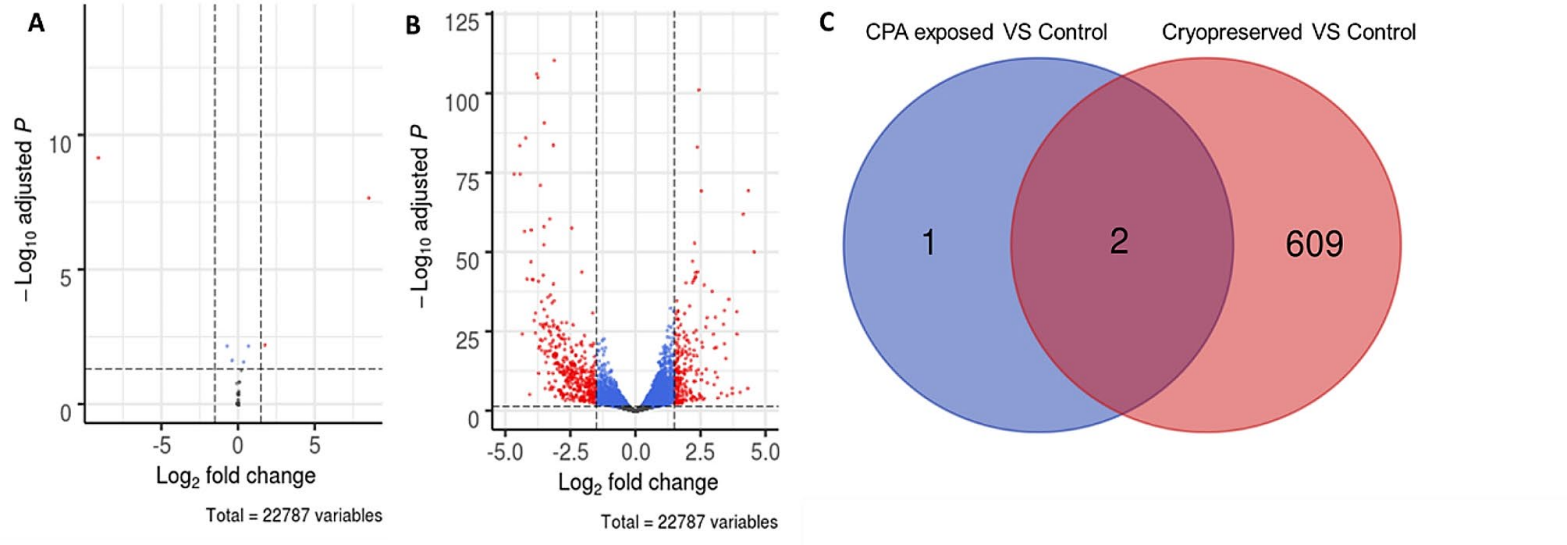
Volcano plots representing the results of differential expression analysis between the two comparisons. **A** Cryoprotectant exposed larvae against fresh larvae. **B** Cryopreserved larvae against fresh larvae. **C** Venn diagram representing the distribution of DEGs between the two comparisons

By using the DESeq2 R package, a total of 22,787 genes were identified among all the samples. In Fig. 1 is showed two volcano plots representing the differentially expressed genes in the two different comparisons: cryoprotectant exposed larvae against fresh larvae (Fig. 1A) and cryopreserved larvae against fresh larvae (Fig. 1B), along with a Venn diagram representing the distribution of DEGs between the two comparisons (Fig. 1C). For the first comparison, 3 DEGs were identified, 1 downregulated and 2 upregulated. Addressing the second comparison, a total of 611 DEGs were found, 378 of them were considered downregulated and the remaining 233 upregulated. Due to the low number of genes differentially expressed in the first condition (Fig. 1A) for further analysis only the second comparison was taken in account.

### Enrichment analysis

For a further understanding of the biological meaning of the DEGs represented under cryopreservation of *C. angulata* larvae, a GO enrichment analysis was performed for all the DEGs, using the Database for Annotation, Visualization and Integrated Discovery (DAVID). The most significantly enriched GO terms in biological processes (BP), cellular components (CC) and molecular functions (MF) were “carbohydrate metabolic process” (GO:0005975), “integral component of membrane” (GO:0016021) and “chitin binding” (GO:0008061), respectively.

**Fig. 2:**
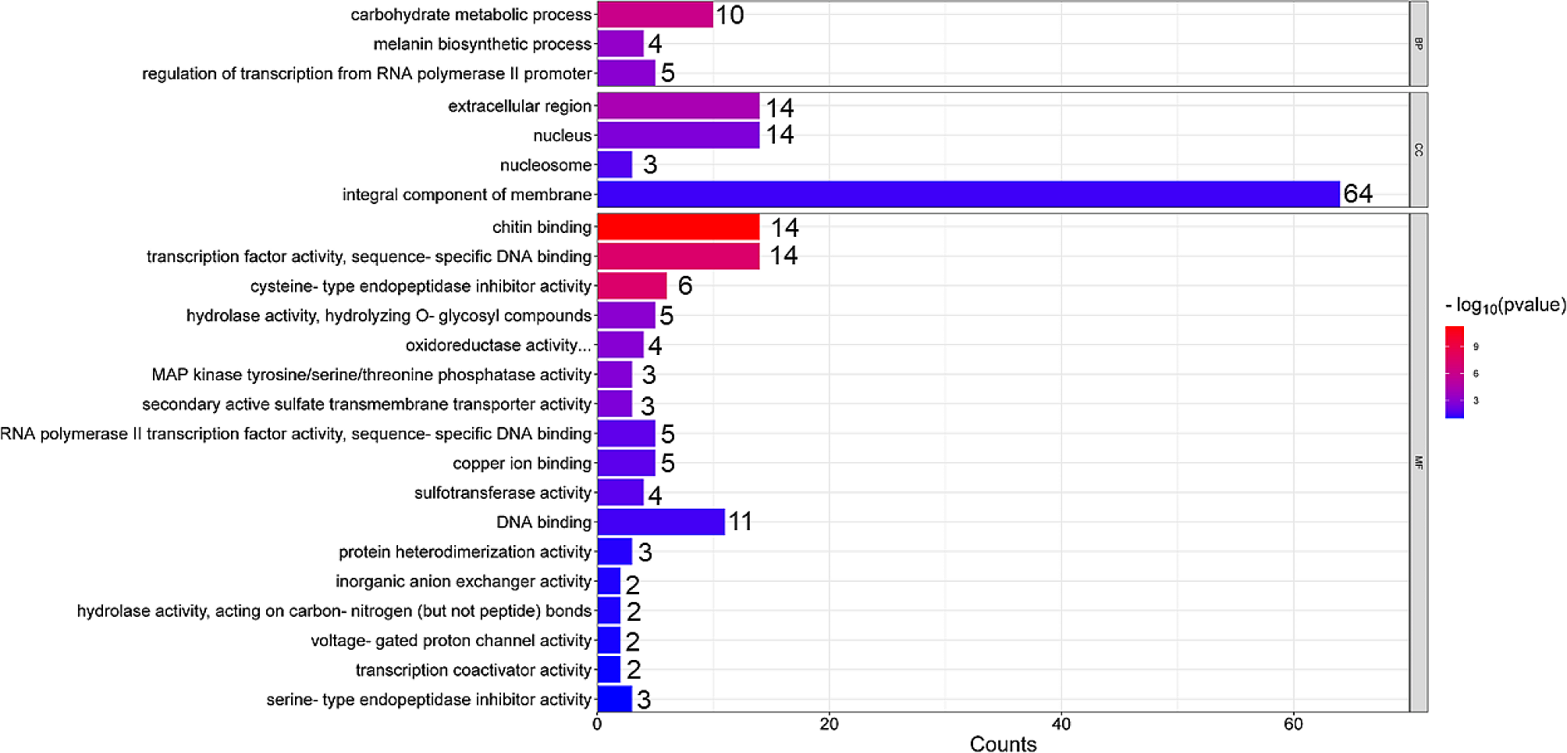
Gene ontology (GO) enrichment results for all differentially expressed genes (DEGs) in the comparison between cryopreserved larvae against fresh larvae

It is also important to mention the significant enrichment of the “extracellular region” (GO:0005576) and the “oxidoreductase activity, acting on paired donors, with incorporation or reduction of molecular oxygen, another compound as one donor, and incorporation of one atom of oxygen” (GO:0016716) (Fig. 2).

**Fig. 3:**
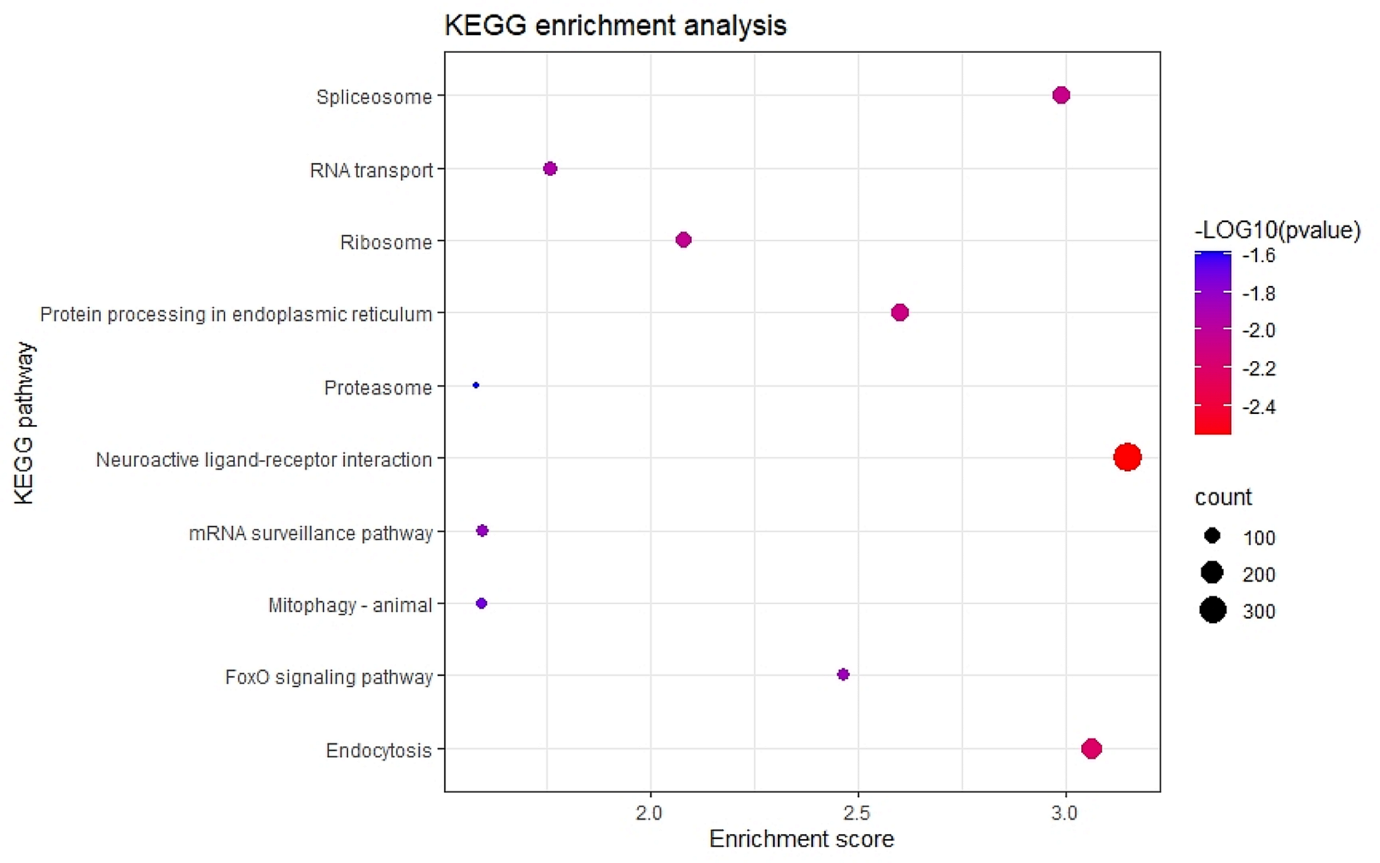
Top 10 enriched pathways according to the results of the KEGG enrichment analysis for all differentially expressed genes in the comparison between cryopreserved larvae against fresh larvae

Moreover, a Kyoto Encyclopedia of Genes and Genomes (KEGG) enrichment analysis for all the DEGs was performed, to assess the most enriched pathways. The 10 most enriched pathways from a total of 126 are represented in Fig. 3. The most enriched pathways were the “neuroactive ligand receptor interaction”, “endocytosis” and “spliceosome”. However, it is also important to notice the significant enrichment of protein regulation pathways such as “protein processing in endoplasmic reticulum” or “proteasome”, and other important developmental pathways as “ribosome” and “FoxO signaling pathway”.

### RT-qPCR confirmation

**Fig. 4:**
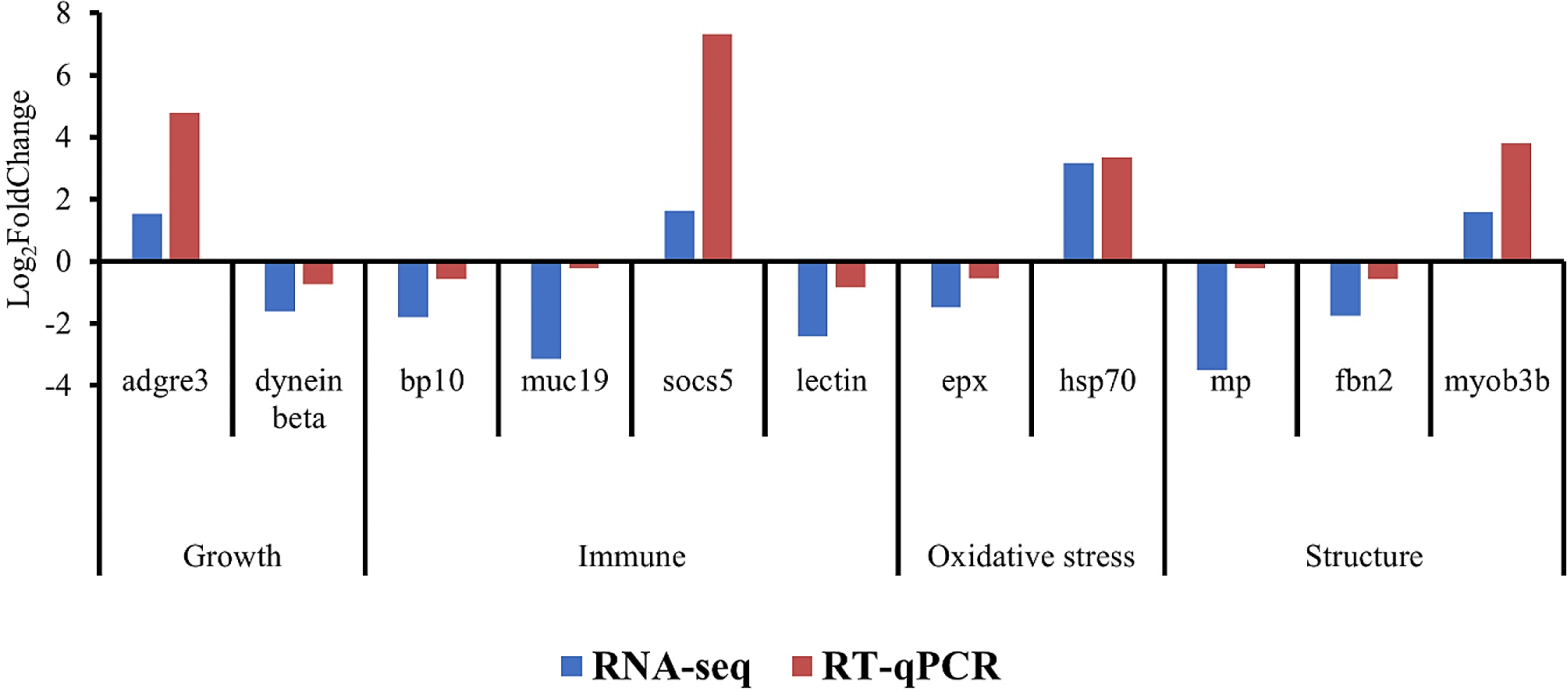
Quantitative qPCR validation of RNA-seq results in terms of relative gene expression

Through RT-qPCR, the results from the RNA-seq analysis were confirmed using a set of 11 genes related to important mechanisms such as growth (*adgre3*, dynein beta), structure (*mp, fbn2, myob3b*), oxidative stress (*epx, hsp70*) and immune response (*bp10, muc19, socs5*, lectin). The comparison between the results of gene expression in both techniques presented the same tendency and for that reason the RNA-seq output was considered to be confirmed. More specifically there was a general upregulation of *adgre3, socs5, hsp70* and *myob3b* regarding control group. Contrarily, dynein beta, *bp10*, *muc19*, lectin, *epx*, *mp* and *fbn2* were downregulated in the two different methods. These results are shown in Fig. 4. Table 3 summarizes the expression results obtain using both methods for all the 11 selected genes, and their respective previously reported function.

**Table 3:** Functions of the differentially expressed genes selected as putative biomarkers of cryodamage. Relative expression and statistical significance are described

Gene name	Gene symbol	RT-qPCR expression	RNA-seq log2Fold change	p_value	Description
adhesion G protein-coupled receptor E3-like	*adgre3*	Up	2.04	2,69e-19	Related to immune function of *Pimephales* sp [85].
dynein beta chain X5	dynein beta	Down	-1.60	9,72e-06	Involved in the generation and regulation of the bending of cilia and flagella in eukaryotes [58, 60] and in the initial shell formation process of *C. gigas* [63]
blastula protease 10	*bp10*	Down	-1.79	4,08e-12	Applied to assess the toxic effects of metals in *P. lividus* embryos [79]
mucin-19	*muc19*	Down	-3.13	1,60e-20	Detected as an immune effector in *Crassostrea virginica* larvae [86]
suppresor of cytokine signaling 5-like	*socs5*	Up	1.63	4,95e-21	Involved in intercellular signal pathways related with the immune system [73, 77], growth and development of the organisms [76]
lectin	lectin	Down	-2.41	6,42e-13	Involved in the self and nonself recognitions, innate immunity, reproduction and food capture and ingestion in bivalves [81–84]
eosinophil peroxidase	*epx*	Down	-2.06	8,33e-23	Protective mechanism of *C. gigas* for mitigating cellular stress and reducing levels of ROS [57]
heat shock protein 70 B2	*hsp70*	Up	3.59	3.87e-06	Considered a stress response in frozen-thawed bovine embryos [53]
mantle protein	*mp*	Down	-4.02	4.66e-42	Protein present in the mantle epithelial cells of bivalves [71]
fibrilin-2	*fbn2*	Down	-1.75	9.19e-12	Down regulated in the mantle and gills of *C. gigas* after hypoxia exposure [72]
myosin-III	*myob3b*	Up	1.78	1.44e-12	Important role in *C. gigas* embryo-larval related to the formation of the muscle structures particularly associated with locomotion [64]

## Discussion

After a few studies in *C. angulata* to establish and improve freezing/thawing protocols, cryopreservation has been identified as a technique with the potential to preserve its genetic lines and enhance its reproduction management [18, 25, 32–34]. Although *C. angulata*, like other bivalves, had promising results in preserving sperm and larvae [18, 25, 32–34], cryopreserving larvae has proven to be challenging due to the multicellular organism’s size and complexity [25]. In bivalves, the evaluation of the post-thaw quality of larvae has been based on swimming activity, morphology, and survival [6, 8, 25]. To elucidate about the molecular changes that occur during the cryopreservation process and identifying the involved molecular mechanisms of cryodamage, the current study has analysed the alterations in gene expression of cryoprotectant exposed and cryopreserved *C. angulata* larvae using RNA sequencing.

### Transcriptomic, functional and enrichment analysis

In the present study it was possible to identify a set of differentially expressed genes in both treatment comparisons. Assessing the comparison between cryoprotectant exposed larvae and fresh larvae, the number of DEGs was considered to be low (2 genes upregulated and 1 downregulated). Therefore, any possible toxic effect dealt by cryoprotectant exposure was not considered for further analysis. However, the number of DEGs detected when comparing cryopreserved larvae against fresh larvae, was considerably higher (233 genes upregulated and 378 downregulated). These results reveals that molecular damage is associated with freezing-thawing process during cryopreservation of *Crassostrea angulata* D-larvae rather than to cryoprotectant exposure.

Following a functional analysis, the relation of all DEGs to specific CC, MF and BP was evaluated through an enrichment analysis. Among these different gene ontologies (GO), the most significantly enriched were integral components of membrane (CC) and Chitin binding (MF). Chitin is the second most abundant natural polysaccharide and it is abundantly present in the shell matrix of oyster species such as *Pinctata fucata martensii* and *C. gigas* [35, 36]. Chitinase and Chitin synthases are vastly represented in the mantle, a very important tissue in shell formation and during early oyster larval development stages [37]. The results of the present study showed a down regulation of chitin synthase, chitin binding proteins and different isoforms of acidic mammalian chitinase in cryopreserved larvae, suggesting an impairment in shell formation and corroborating the data presented by Anjos et al. [25] in previous studies. Also, chitinases are known for their importance in immune response and by helping to maintain normal life cycle functions since, not only synthesis, but also degradation of chitin, are important in the development of chitin containing organisms [38]. Different DNA binding related molecular functions (RNA polymerase II transcription factor activity, sequence specific DNA binding; transcription factor activity, sequence-specific DNA binding; DNA binding) were also significantly enriched. An example of DNA binding functions are the changes occurring in DNA methylation during early development in *C. gigas* as reported by Riviere et al. [39]. Moreover, Zhao et al. [40] hypothesized that due to its connection to cell proliferation, DNA binding was a key element in stress response. This means that as a stress factor, the cryopreservation process may induce an impairment in the early development of *C. angulata* larvae.

Regarding the KEGG pathway enrichment analysis, the most significantly enriched pathway was the “neuroactive ligand-receptor interaction”. This pathway is made up of receptors situated on plasma membranes that are involved in signal transduction from the external environment into cells [41]. An analysis of the transcriptome of *C. gigas* and *P.f. martensii* at different stages of development revealed that neuroendocrine pathways such as this one, were implicated in important developmental functions like shell formation, settling, and metamorphosis [42]. Furthermore, a previous study by Lu et al. [43] reported a significant enrichment of this pathway upon induced stress in *P.f. martensii*. Both previous studies and the results of our study are a plausible evidence of the malformations observed by Anjos et al. [25].

The second two most significant enriched pathways were the endocytosis and the spliceosome. The first one is an imperative part of the membrane receptors activity and quantity control, regulating the signal transduction mediated by those receptors [44]. This pathway is also known to be related to cell proliferation and organism growth since it is connected to the degradation of epidermal growth factor receptors (EGFR) [45]. These receptors were referred to be directly corelated to the development and growth of the pearl oyster [46]. Also, Li et al. [47], proposed that endocytosis-induced processes such as signal transduction and plasma membrane proteins degradation, may regulate oyster growth by integrating endogenous signaling pathways and environmental input. These previous studies corroborate the results of the present study and therefore, it is hypothesized that cryopreservation may affect growth in *C. angulata* larvae.

Concerning “spliceosome”, as a key tool of genetic information processing, it is extremely important in the survival, adaptation and development of the organism [48]. Previous studies have already demonstrated an impairment of this process due to stress factors such as ocean acidification [49]. The same may be assumed by observing the functional analysis results of the present research, more specifically, the significant enrichment of processes like “DNA binding”.

In our study, there was also a significant enrichment of the “ribosome” pathway. This molecular organelle is responsible for the translational capacity of a cell and so, directly related to protein synthesis and functions such as cell growth, proliferation and apoptosis [50]. Moreover, other protein processing related pathways were significantly enriched, the “proteasome” and “protein processing in endoplasmic reticulum”, suggesting an increased amount of degraded proteins. Cryopreservation clearly induces a stress response in this organism and apparently leads to a development malfunction, mainly at the shell formation and membrane level.

### Putative biomarkers of cryopreserved larval quality

To validate RNAseq data, a selection of 11 genes was done according to their relevance in certain functions. These genes were checked for their putative use as biomarkers of post-thaw larval quality. One of the functions affected by cryopreservation was the oxidative stress system. Heat shock proteins 70, *hsp70*, a subgroup of chaperones, are important to maintain the homeostasis of the cell and has the capacity of counteract apoptotic mechanisms, interviewing in cell processes such as cell movement and cytoskeleton stabilization [51, 52]. Our findings indicate that the expression levels of *hsp70* were higher in post-thaw D-larvae than in fresh larvae (larvae dilute in FSW). This result is in line with data reported by Park et al. [53], where the expression levels of four apoptotic-related genes, including *hsp70*, were observed to be significantly elevated in cryopreserved bovine embryos. This expression may arise, as suggested by the authors, as a stress response and potentially compromised developmental ability. Overall, these proteins have the function of preventing thermal or oxidative stress, which is commonly associated with cryopreservation procedures.

Eosinophil peroxidase (*epx*) is an enzyme released from eosinophils granulocytes, that is essential to maintain the main function and homeostasis of eosinophils [54]. Eosinophils are made part of a group of cells known as hemocytes that play a crucial role in bivalve immune response to defend against different stressors, such as pathogens, temperature, acidification and pollution [55]. In the present work, the *epx* expression revealed that this gene was suppressed in D-larvae after freezing/thawing steps. Other studies, conducted in *Anguilla japonica* and *C. gigas* reported that when organisms were exposed to osmotic stress conditions, the levels of *epx* were also suppressed [56, 57], having been suggested by Zhao et al. [57] that this could serve as a protective tactic for mitigating cellular stress and lowering levels of reactive oxygen species (ROS). The observed suppression of *epx* expression in our study may be related to our experimental protocol that involved environmental fluctuations due to the freezing/thawing process, which must have induced thermal stress, leading to increased expression of *hsp70* rather than *epx*.

The dynein beta chain flagellar outer arm, *dyh4*, belongs to the dynein protein family. Dynein proteins are divided into two main groups, which are cytoplasmic and axonemal dynein [58]. Cytoplasmic dynein is responsible for intracellular transport and cell mitosis [58, 59]. Axonemal dynein is a microtubule-based molecular motor that is in charge of the generation and regulation of the bending of cilia and flagella in eukaryotes [58, 60]. Dynein arms convert the chemical energy released upon ATP binding into mechanical force, producing the driving power for the organelles’ movement [60]. Cilia and flagella play important roles in bivalve sperm and larvae motility. The expression of dynein beta in thawed *C. angulata* D-larvae was lower than in the fresh larvae, suggesting some level of cilia impairment which may affect the larvae swimming performance and, in further larvae development stages, their feeding behavior and sensorial role. Unfortunately in the present study we could not follow larval development but this data corroborated previous findings reported by our group, where a significant lower velocity and motility was observed in thawed *C. angulata* D-larvae when compared with the control group [25]. Similar findings were reported by Suquet et al. [61] and Suneja et al. [62], where lower performances in swimming activity of *C. gigas* post-thaw D-larvae were reported. Suneja et al. [62] even explored the organogenesis of these larvae and identified development problems in the velum structure as a result of cryodamage, which suggests that modifications in the structure of the velum can lead to cilia impairment. This can affect the swimming and feeding behavior and compromise the performance of the larvae or even lead to their death at later stages. Apart from impairing structural modification associated with filtration and movement mechanisms, the downregulation of dynein motor proteins may also contribute to shell malformations, previously detected Anjos et al. [25] since as stated by De Wit et al. [63] these proteins have an important role as transporters of cellular components during the initial stages of shell formation in this species.

Myosin is the key muscle protein of thick filaments [64] and its function is to transform chemical energy in mechanical force that travels along actin filaments, resulting in the contraction of the muscle [65]. In bivalves, during larval development, the actin filaments or expression profiles of myosin heavy chain are mainly found in the velum retractor and adductor muscle, while in the adult phase in the adductor and mantle muscles [64, 66, 67]. Myosin heavy chain seems to have an important role in *C. gigas* embryo-larval developmental phases, especially in the stages of trochophore and D-larvae, due to the formation of the muscle structures during embryogenesis some associated with larval locomotion [64]. Thus, our results revealed that cryopreservation may have affected certain mechanisms in *C. angulata* D-larvae related to myosin protein production. This is evidenced by the higher gene expression of *myob3b* in the cryopreserved D-larvae compared to the control group, which may suggest a compensatory response to the cryopreservation process.

Fibrillin-2 is a protein encoded by the *fbn2* gene that belongs to the fibrillin family [68]. In *C. gigas*, fibrillin is highly expressed in the mantle [69], a soft tissue layer that lines the inner shell and covers the visceral mass. This structure provides protection, contributes to shell formation, facilitates respiration and plays a crucial role in the oyster’s filter feeding mechanism [70, 71]. Our results showed that expression of the *fbn2* transcript was suppressed after cryopreservation. This suggests that there may be an alteration at the level of the extracellular matrix of *C. angulata* larvae. This alteration is likely to have implications in the development and maintenance of tissues, potentially jeopardizing the subsequent larval developmental stages. David et al. [72] observed a down-regulation of the fibrillin gene in the mantle and gills of *C. gigas* after 24 days of hypoxia exposure. These results together with the ones obtained for *mp*, a specific mantle protein present in the epithelial cells, where expression was also suppressed, demonstrates that these alterations could compromise proper functioning, which can lead to alterations in the mantle tissues, shell formation and feeding capacity, especially during the early stages of development when structures are being formed. Some morphological alterations as mantle protuberance and reduction of body size related to the shell were detected in *C. angulata* post-thaw D-larvae in a prior work conducted by our group [25]. These morphological alterations can be related to *mp* gene suppression observed after freezing/thawing steps.

Suppressors of cytokine signaling (*socs*) are a class of inhibitory proteins that negatively regulate cytokine signal transduction [73, 74]. These inhibitory proteins play an essential role in several intercellular signal pathways that are engaged in the immune system [73, 75], growth and development of the organisms [76] being identified in several organs of *C. gigas* [75]. In our results, we identified the *socs5* gene as being upregulated in cryopreserved larvae when compared with the fresh larvae. This result suggested that the cryopreservation protocol may have induced physical stress in the D-larvae of *C. angulata*, due to exposure to a combination of hyperosmotic solution (cryoprotectant solution) with temperature variations (freezing/thawing steps). These stress factors may have activated the production of cytokines, and to control an excessive response that can be harmful to the host, *socs5* was activated. Similar results were obtained by De Zoysa et al. [77] with the *socs2* gene being upregulated in *Haliotis discus discus* during thermal, low-salinity and hypoxic stress.

Blastula protease 10, *bp10*, involved in immune functions and embryonic development [78, 79] is an enzyme that belongs to the astacin metalloprotease family [80]. This gene has been used to evaluate the toxic effects of metals and other contaminants in *Paracentrotus lividus* embryos being upregulated or downregulated depending on the compound tested [79]. Our results revealed that cryopreservation suppressed the *bp10* expression in the D-larvae of *C. angulata* when compared to the control. However, little is known about the *bp10* gene in oysters and further studies will be needed to characterize and understand how variation in the expression of this metal binding protein is affected in cryopreserved larvae.

Lectins play a fundamental role in the self and nonself recognitions, innate immunity, reproduction and food capture and ingestion in bivalves [81–84]. Particularly in shell formation, these group of proteins are involved in the extracellular matrix agglutination [63]. In our study, the expression of lectins in *C. angulata* cryopreserved D-larvae was lower than in the fresh larvae. Since lectins can bind carbohydrates present in the surface of microalgae, they participate in the recognitions of food particles. Therefore, in our study changes in lectin gene expression may affect larvae’s digestive functions, impairing their ability to efficiently capture and process food. It is crucial to understand the implications of altered lectin gene expression in *C. angulata* thawed D-larvae as it can have consequences on their feeding selection and further digestive function and ultimately in larval developmental outcomes.

## Conclusions

In this study, the analysis of differential gene expression revealed significant changes in genes associated with growth, structural development, oxidative stress response, and the immune system in cryopreserved *C. angulata* larvae, compared to fresh larvae. No effects were seen in larvae exposed to cryoprotectants. These findings underscore the importance of considering both molecular and physiological aspects in the development of reliable cryopreservation protocols and in the detection of relevant biomarkers of cryodamage in *C. angulata* D-larvae. Furthermore, they highlight the critical step in the cryopreservation process, revealing that gene expression is not affected by cryoprotectant exposure as it is by the freezing process itself. Importantly, this study emphasizes the significance of our findings for the shellfish sector, providing valuable insights for future research in cryobiology and support aquaculture and restocking programs.

## Data Availability

The datasets generated and analysed during the current study are available in the Gene Expression Omnibus (GEO) repository, with accession number GSE246924 to datasets.

## Abbreviations

NGS: Next-generation sequencing
RNA-seq: RNA sequencing
CPAs: Cryoprotectant solution
FSW: Filtered and UV- sterilized seawater
CASA: Computer-assisted sperm analysis
DMSO: Dimethyl sulfoxide
PVP: Polyvinylpyrrolidone
LN: Liquid nitrogen
GO: Gene ontology
FDR: False Discovery Rate
FC: Fold Change
GSEA: Gene Set Enrichment Analysis
DEGs: Differentially expressed genes
RT-qPCR: Quantitative RT-PCR
DAVID: Database for Annotation, Visualization and Integrated Discovery
BP: Biological processes
CC: Cellular components
MF: Molecular functions
KEGG: Kyoto Encyclopedia of Genes and Genomes
ROS: Reactive oxygen species
EGFR: Epidermal growth factor receptors

## References

[1] Wang H, Qian L, Liu X, Zhang G, Guo X (2010). Classification of a common cupped oyster from Southern China. J Shellfish Res.

[2] Grizel H, Héral M (1991). Introduction into France of the Japanese oyster (Crassostrea gigas). ICES J Mar Sci.

[3] Huvet A, Lapègue S, Magoulas A, Boudry P (2000). Mitochondrial and nuclear DNA phylogeography of Crassostrea angulata, the Portuguese oyster endangered in Europe. Conserv Genet.

[4] Anjos C, Baptista T, Joaquim S, Mendes S, Matias AM, Moura P (2017). Broodstock conditioning of the Portuguese oyster (Crassostrea angulata, Lamarck, 1819): influence of different diets. Aquac Res.

[5] Adams SL, Smith JF, Roberts RD, Janke AR, King NG, Tervit HR (2008). Application of sperm cryopreservation in selective breeding of the Pacific oyster, Crassostrea gigas (Thunberg). Aquac Res.

[6] Paredes E (2015). Exploring the evolution of marine invertebrate cryopreservation – landmarks, state of the art and future lines of research. Cryobiology.

[7] Martínez-Páramo S, Horváth Á, Labbé C, Zhang T, Robles V, Herráez P (2017). Cryobanking of aquatic species. Aquaculture.

[8] Yang H, Huo Y (2022). Review of molluscan larval cryopreservation and application to germplasm cryobanking and commercial seed production. Aquaculture.

[9] Labbé C, Haffray P, Mingant C, Quittet B, Diss B, Tervit HR (2018). Cryopreservation of Pacific oyster (*Crassostrea gigas*) larvae: revisiting the practical limitations and scaling up the procedure for application to hatchery. Aquaculture.

[10] Suquet M, Labbé C, Puyo S, Mingant C, Quittet B, Boulais M (2014). Survival, Growth and Reproduction of Cryopreserved Larvae from a Marine Invertebrate, the Pacific Oyster (*Crassostrea gigas*). PLoS ONE.

[11] Robles V, Cabrita E, Acker JP, Herráez P. Embryo cryopreservation: what we know until now. In: Cabrita E, Robles V, Herráez P, editors. Methods in reproductive aquaculture: marine and freshwater species. CRC Press; 2008. p. 265–94.

[12] Wagh V, Meganathan K, Jagtap S, Gaspar JA, Winkler J, Spitkovsky D (2011). Effects of Cryopreservation on the transcriptome of human embryonic stem cells after thawing and culturing. Stem Cell Reviews Rep.

[13] Nynca J, Arnold GJ, Fröhlich T, Ciereszko A (2015). Cryopreservation-induced alterations in protein composition of rainbow trout semen. Proteomics.

[14] Ciereszko A, Dietrich MA, Nynca J (2017). Fish semen proteomics — new opportunities in fish reproductive research. Aquaculture.

[15] Yang Y, Liu D, Wu L, Huang W, Yang S, Xia J (2019). Comparative transcriptome analyses reveal changes of gene expression in fresh and cryopreserved yellow catfish (*Pelteobagrus fulvidraco*) sperm and the effects of Cryoprotectant Me_2_SO. Int J Biol Macromol.

[16] Wang Z, Gerstein M, Snyder M (2009). RNA-Seq: a revolutionary tool for transcriptomics. Nat Rev Genet.

[17] Metzker ML (2010). Sequencing technologies the next generation. Nat Rev Genet.

[18] Riesco MF, Félix F, Matias D, Joaquim S, Suquet M, Cabrita E (2019). Comparative study on cellular and molecular responses in oyster sperm revealed different susceptibilities to cryopreservation. Aquaculture.

[19] Hossen S, Sukhan ZP, Cho Y, Kho KH (2021). Effects of cryopreservation on gene expression and post thaw sperm quality of Pacific Abalone, Haliotis discus hannai. Front Mar Sci.

[20] Liu Y, Catalano SR, Qin J, Han J, Zhan X, Li X (2022). Effects of cryopreservation on redox status and gene expression of trochophore larvae in Mytilus galloprovincialis. J World Aquaculture Soc.

[21] Liu Y, Zhan X, Catalano SR, Qin J, Han J, Li X (2022). Investigation on redox status and gene expression related to larval cryopreservation in the Pacific oyster Crassostrea gigas. Fish Sci.

[22] Zhang J, Tian Y, Wang L, Li Z, Wu Y, Li Z, et al. Comparative transcriptome analyses reveal changes of gene expression in larvae hatched from fresh and cryopreserved kelp grouper (*Epinephelus moara*) embryos. Aquaculture. 2022;547:737459.

[23] Wang H, Montague HR, Hess HN, Zhang Y, Aguilar GL, Dunham RA, et al. Transcriptome analysis reveals key gene expression changes in Blue Catfish sperm in response to Cryopreservation. Int J Mol Sci. 2022;23:7618. 10.3390/ijms23147618 PMC9316979 35886966

[24] Niu J, Wang X, Liu P, Liu H, Li R, Li Z, et al. Effects of cryopreservation on sperm with cryodiluent in Viviparous Black Rockfish (*Sebastes schlegelii*). Int J Mol Sci. 2022;23:3392. 10.3390/ijms23063392 PMC8955014 35328812

[25] Anjos C, Duarte D, Diogo P, Matias D, Cabrita E (2022). Assessment of larval quality of two bivalve species, Crassostrea angulata and Chamelea gallina, exposed and cryopreserved with different cryoprotectant solutions. Cryobiology.

[26] Kopylova E, Noé L, Touzet H (2012). SortMeRNA: Fast and accurate filtering of ribosomal RNAs in metatranscriptomic data. Bioinformatics.

[27] Qi H, Cong R, Wang Y, Li L, Zhang G (2023). Construction and analysis of the chromosome-level haplotype-resolved genomes of two Crassostrea oyster congeners: Crassostrea angulata and Crassostrea gigas. GigaScience.

[28] Götz S, García-Gómez JM, Terol J, Williams TD, Nagaraj SH, Nueda MJ (2008). High-throughput functional annotation and data mining with the Blast2GO suite. Nucleic Acids Res.

[29] Patro R, Duggal G, Love MI, Irizarry RA, Kingsford C (2017). Salmon provides fast and bias-aware quantification of transcript expression. Nat Methods.

[30] Love MI, Huber W, Anders S (2014). Moderated estimation of Fold change and dispersion for RNA-seq data with DESeq2. Genome Biol.

[31] Livak KJ, Schmittgen TD (2001). Analysis of relative gene expression data using real-time quantitative PCR and the 2-∆∆CT method. Methods.

[32] Riesco MF, Félix F, Matias D, Joaquim S, Suquet M, Cabrita E (2017). First study in cryopreserved Crassostrea angulata sperm. Gen Comp Endocrinol.

[33] Anjos C, Santos AL, Duarte D, Matias D, Cabrita E (2021). Effect of Trehalose and sucrose in Post-thaw Quality of Crassostrea angulata sperm. Front Physiol.

[34] Kuo TY, Gwo JC. Quality assessment of cryopreserved Portuguese oyster (*Crassostrea angulata*) sperm through ultrastructural and flow cytometry analysis. Cryobiology. 2022;104:79–86. 10.1016/j.cryobiol.2021.09.005 34537223

[35] Suzuki M, Sakuda S, Nagasawa H (2007). Identification of chitin in the prismatic layer of the shell and a chitin synthase gene from the Japanese pearl oyster, Pinctada fucata. Bioscience Biotechnol Biochem.

[36] Lee SW, Choi CS. The correlation between organic matrices and biominerals (myostracal prism and folia) of the adult oyster shell, *Crassostrea gigas*. Micron. 2007;38:58–64. 10.1016/j.micron.2006.03.018 16757172

[37] Du X, Fan G, Jiao Y, Zhang H, Guo X, Huang R (2017). The pearl oyster *Pinctada Fucata martensii* genome and multi-omic analyses provide insights into biomineralization. GigaScience.

[38] Badariotti F, Thuau R, Lelong C, Dubos MP, Favrel P (2007). Characterization of an atypical family 18 chitinase from the oyster Crassostrea gigas: evidence for a role in early development and immunity. Dev Comp Immunol.

[39] Riviere G, Wu GC, Fellous A, Goux D, Sourdaine P, Favrel P (2013). DNA methylation is crucial for the Early Development in the Oyster C. Gigas. Mar Biotechnol.

[40] Zhao X, Yu H, Kong L, Li Q. Transcriptomic responses to salinity stress in the Pacific Oyster *Crassostrea gigas*. PLoS ONE. 2012;7:e46244. 10.1371/journal.pone.0046244 PMC3459877 23029449

[41] Lauss M, Kriegner A, Vierlinger K, Noehammer C (2007). Characterization of the drugged human genome. Pharmacogenomics.

[42] Zheng Z, Hao R, Xiong X, Jiao Y, Deng Y, Du X (2019). Developmental characteristics of pearl oyster Pinctada fucata martensii: insight into key molecular events related to shell formation, settlement and metamorphosis. BMC Genomics.

[43] Lu J, Zhang M, Liang H, Shen C, Zhang B, Liang B (2022). Comparative proteomics and transcriptomics illustrate the allograft-induced stress response in the pearl oyster (Pinctada Fucata martensii). Fish Shellfish Immunol.

[44] Yarden Y, Tarcic G. Vesicle trafficking in cancer. 1st ed. Springer: New York; 2013.

[45] Wu M, Zhang P. EGFR-mediated autophagy in tumourigenesis and therapeutic resistance. Cancer Lett. 2020;469:207–16. 10.1016/j.canlet.2019.10.030 31639425

[46] Wang Q, Hao R, Zhao X, Huang R, Zheng Z, Deng Y (2018). Identification of EGFR in pearl oyster (Pinctada Fucata martensii) and correlation analysis of its expression and growth traits. Bioscience Biotechnol Biochem.

[47] Li Y, Yang B, Shi C, Tan Y, Ren L, Mokrani A (2023). Integrated analysis of mRNAs and lncRNAs reveals candidate marker genes and potential hub lncRNAs associated with growth regulation of the Pacific Oyster, Crassostrea gigas. BMC Genomics.

[48] Mitra T, Mahanty A, Ganguly S, Mohanty BP (2020). Transcriptomic responses to pollution in natural riverine environment in Rita rita. Environ Res.

[49] Wang X, Li P, Cao X, Liu B, He S, Cao Z, et al. Effects of ocean acidification and tralopyril on bivalve biomineralization and carbon cycling: a study of the Pacific Oyster (*Crassostrea gigas*). Environ Pollut. 2022;313:120161. 10.1016/j.envpol.2022.120161 36100119

[50] Liu Q, Shvarts T, Sliz P, Gregory RI (2020). RiboToolkit: an integrated platform for analysis and annotation of ribosome profiling data to decode mRNA translation at codon resolution. Nucleic Acids Res.

[51] Balogi Z, Multhoff G, Jensen TK, Lloyd-Evans E, Yamashima T, Jäättelä M (2019). Hsp70 interactions with membrane lipids regulate cellular functions in health and disease. Prog Lipid Res.

[52] Zhang G, Fang X, Guo X, Li L, Luo R, Xu F (2012). The oyster genome reveals stress adaptation and complexity of shell formation. Nature.

[53] Park SY, Kim EY, Cui XS, Tae JC, Lee WD, Kim NH (2006). Increase in DNA fragmentation and apoptosis-related gene expression in frozen-thawed bovine blastocysts. Zygote.

[54] Choi KM, Joo MS, Kang G, Woo WS, Kim KH, Jeong SH (2021). First report of eosinophil peroxidase in starry flounder (Platichthys stellatus): gene identification and gene expression profiling. Fish Shellfish Immunol.

[55] de la Ballina NR, Maresca F, Cao A, Villalba A. Bivalve haemocyte subpopulations: a review. Front Immunol. 2022;13:826255. 10.3389/fimmu.2022.826255 PMC9024128 35464425

[56] Tse WKF, Sun J, Zhang H, Law AYS, Yeung BHY, Chow SC (2013). Transcriptomic and iTRAQ proteomic approaches reveal novel short-term hyperosmotic stress responsive proteins in the gill of the Japanese eel (Anguilla japonica). J Proteom.

[57] Zhao X, Yu H, Kong L, Li Q (2016). Gene Co-expression Network Analysis reveals the correlation patterns among genes in Euryhaline Adaptation of Crassostrea gigas. Mar Biotechnol.

[58] Toda A, Nishikawa Y, Tanaka H, Yagi T, Kurisu G (2020). The complex of outer-arm dynein light chain-1 and the microtubule-binding domain of the γ heavy chain shows how axonemal dynein tunes ciliary beating. J Biol Chem.

[59] Canty JT, Tan R, Kusakci E, Fernandes J, Yildiz A (2021). Structure and Mechanics of Dynein Motors. Annual Rev Biophys.

[60] Aprea I, Raidt J, Höben IM, Loges NT, Nöthe-Menchen T, Pennekamp P, et al. Defects in the cytoplasmic assembly of axonemal dynein arms cause morphological abnormalities and dysmotility in sperm cells leading to male infertility. 2021;17:e1009306. 10.1371/journal.pgen.1009306 PMC7909641 33635866

[61] Suquet M, Le Mercier A, Rimond F, Mingant C, Haffray P, Labbé C (2012). Setting tools for the early assessment of the quality of thawed Pacific oyster (Crassostrea gigas) D-larvae. Theriogenology.

[62] Suneja S, Alfaro A, Rusk A, Morrish J, Tervit H, McGowan L (2014). Multi-technique approach to characterise the effects of cryopreservation on larval development of the Pacific oyster (Crassostrea gigas). N Z J Mar Freshwat Res.

[63] De Wit P, Durland E, Ventura A, Langdon CJ (2018). Gene expression correlated with delay in shell formation in larval Pacific oysters (Crassostrea gigas) exposed to experimental ocean acidification provides insights into shell formation mechanisms. BMC Genomics.

[64] Li H, Yu H, Li Q (2021). Striated myosin heavy chain gene is a crucial regulator of larval myogenesis in the pacific oyster Crassostrea gigas. Int J Biol Macromol.

[65] Asai DJ, Wilkes DE (2004). The Dynein Heavy Chain Family. J Eukaryot Microbiol.

[66] Li H, Li Q, Yu H, Du S (2019). Developmental dynamics of myogenesis in Pacific oyster Crassostrea gigas. Comp Biochem Physiol Part - B: Biochem Mol Biology.

[67] Yang Y, Zhou L, Yu T, Zheng Y, Wu B, Liu Z (2023). Molecular characterization of myosin heavy chain and its involvement in muscle growth and development in the Yesso scallop Patinopecten Yessoensis. Aquaculture.

[68] Zhang H, Apfelroth SD, Hu W, Davis EC, Sanguineti C, Bonadio J (1994). Structure and expression of fibrillin-2, a novel microfibrillar component preferentially located in elastic matrices. J Cell Biol.

[69] Miyamoto H, Hamaguchi M, Okoshi K. Analysis of genes expressed in the mantle of oyster *Crassostrea gigas*. Fish Sci. 2002;68:651–8.

[70] Joubert C, Linard C, Le Moullac G, Soyez C, Saulnier D, Teaniniuraitemoana V (2014). Temperature and food influence shell growth and mantle gene expression of shell matrix proteins in the pearl oyster *Pinctada margaritifera*. PLoS ONE.

[71] Yarra T, Gharbi K, Blaxter M, Peck LS, Clark MS (2016). Characterization of the mantle transcriptome in bivalves: Pecten maximus, Mytilus edulis and Crassostrea gigas. Mar Genom.

[72] David E, Tanguy A, Pichavant K, Moraga D (2005). Response of the Pacific oyster Crassostrea gigas to hypoxia exposure under experimental conditions. FEBS J.

[73] De Zoysa M, Lee J (2009). Suppressor of cytokine signaling 2 (SOCS-2) homologue in disk abalone: Cloning, sequence characterization and expression analysis. Fish Shellfish Immunol.

[74] Liu WG, Huang X, De, Wang Q, Zhao M, Wu SZ, He MX (2013). Gene cloning and function analysis of cytokine-induced suppressor of cytokine signaling (SOCS) from pearl oyster Pinctada Fucata. Fish Shellfish Immunol.

[75] Li J, Zhang Y, Zhang Y, Liu Y, Xiang Z, Qu F (2015). Cloning and characterization of three suppressors of cytokine signaling (SOCS) genes from the Pacific oyster, Crassostrea gigas. Fish Shellfish Immunol.

[76] Ilangumaran S, Ramanathan S, Rottapel R (2004). Regulation of the immune system by SOCS family adaptor proteins. Semin Immunol.

[77] De Zoysa M, Whang I, Lee Y, Lee S, Lee JS, Lee J (2009). Transcriptional analysis of antioxidant and immune defense genes in disk abalone (Haliotis discus discus) during thermal, low-salinity and hypoxic stress. Comp Biochem Physiol - B Biochem Mol Biology.

[78] Hartman R, Pales Espinosa E, Allam B (2018). Identification of clam plasma proteins that bind its pathogen Quahog Parasite unknown. Fish Shellfish Immunol.

[79] Bonaventura R, Costa C, Deidda I, Zito F, Russo R. Gene expression analysis of the stress response to Lithium, Nickel, and Zinc in *Paracentrotus lividus* embryos. Toxics. 2022;10:325. 10.3390/toxics10060325 PMC9231221 35736933

[80] Lhomond G, Ghiglione C, Lepage T, Gache C (1996). Structure of the gene encoding the sea urchin blastula protease 10 (BP10), a member of the astacin family of Zn2+-metalloproteases. Eur J Biochem.

[81] Allam S, Allam B, Pales Espinosa E. Regulation of mucosal lectins in the oyster *Crassostrea virginica* in response to food availability and environmental factors. J Molluscan Stud. 2021;87:eyaa037.

[82] Pales Espinosa E, Allam B (2013). Food quality and season affect gene expression of the mucosal lectin MeML and particle sorting in the blue mussel Mytilus edulis. Mar Biol.

[83] Wang W, Gong C, Han Z, Lv X, Liu S, Wang L (2019). The lectin domain containing proteins with mucosal immunity and digestive functions in oyster Crassostrea gigas. Fish Shellfish Immunol.

[84] Saco A, Suárez H, Novoa B, Figueras A. A genomic and transcriptomic analysis of the C-Type lectin Gene Family reveals highly expanded and Diversified repertoires in Bivalves. Mar Drugs. 2023;21:254. 10.3390/md21040254 PMC10140915 37103393

[85] Klymus KE, Hrabik RA, Thompson NL, Cornman RS (2022). Genome resequencing clarifies phylogeny and reveals patterns of selection in the toxicogenomics model Pimephales promelas. PeerJ.

[86] Modak TH, Gomez-Chiarri M (2020). Contrasting immunomodulatory effects of probiotic and pathogenic bacteria on eastern oyster, Crassostrea virginica, larvae. Vaccines.

